# Vertebrate Evolution Conserves Hindbrain Circuits despite Diverse Feeding and Breathing Modes

**DOI:** 10.1523/ENEURO.0435-20.2021

**Published:** 2021-04-16

**Authors:** Shun Li, Fan Wang

**Affiliations:** Department of Neurobiology, Duke University, Durham, NC 27710

**Keywords:** breathing, central rhythm generator, evolution, feeding, hindbrain

## Abstract

Feeding and breathing are two functions vital to the survival of all vertebrate species. Throughout the evolution, vertebrates living in different environments have evolved drastically different modes of feeding and breathing through using diversified orofacial and pharyngeal (oropharyngeal) muscles. The oropharyngeal structures are controlled by hindbrain neural circuits. The developing hindbrain shares strikingly conserved organizations and gene expression patterns across vertebrates, thus begs the question of how a highly conserved hindbrain generates circuits subserving diverse feeding/breathing patterns. In this review, we summarize major modes of feeding and breathing and principles underlying their coordination in many vertebrate species. We provide a hypothesis for the existence of a common hindbrain circuit at the phylotypic embryonic stage controlling oropharyngeal movements that is shared across vertebrate species; and reconfiguration and repurposing of this conserved circuit give rise to more complex behaviors in adult higher vertebrates.

## Significance Statement

Understanding how a highly conserved hindbrain generates diverse feeding/breathing patterns is important for elucidating neural mechanisms underlying the execution and coordination of these two vital behaviors. Here, we first briefly summarize key modes of vertebrates feeding/breathing, discuss main principles coordinating feeding/breathing, and provide a unifying hypothesis for the existence of a shared oropharyngeal movement control circuit across species. By synthesizing behavior, structural and neural mechanisms for feeding/breathing functions across evolution, we believe that this review and our hypothesis can open new research avenues for elucidating the precise hindbrain circuits controlling feeding, breathing, and other oropharyngeal functions.

## Introduction

The orofacial and pharyngeal regions of vertebrates, derived from pharyngeal arches (PAs) in the embryo, are critical for executing the two key survival functions, breathing and feeding. Vertebrates share a highly conserved embryonic hindbrain organization, both in terms of gene expression profiles and stereotyped rhombomere arrangement (Kiecker and Lumsden, 2005; Parker and Krumlauf, 2020). Yet, they use widely different approaches for feeding and breathing, that rely on a diversity of oropharyngeal structures and muscles (Bels and Whishaw, 2019). In this review, we first summarize major modes of feeding and breathing and then examine different manners through which these two actions are coordinated throughout vertebrate evolution. Finally, we provide a hypothesis for how a highly conserved embryonic hindbrain can assemble neural circuits that control drastically different feeding and breathing apparatuses and behaviors across species. Important terminologies are listed and defined in Table 1.

**Table 1:** Glossary

Buccal cavity	Anterior portion of the digestive system that is bounded by the lips anteriorly and palatoglossal arch posteriorly.
Pharynx	Part of the throat posterior to the oral and nasal cavity, sitting above the esophagus.
Larynx	An organ that sits at the anterior neck, gating the entrance of trachea/lung and housing the vocal fold for vocalization.
Pharyngeal slits	Series of openings in the pharynx. Originally assisted in filter-feeding in primitive chordates and have been modified extensively throughout evolution.
Hyolingual apparatus	The hyoid (a U-shaped bone at the anterior neck anchoring the tongue and larynx) and tongue are collectively referred to as the hyolingual apparatus.
Glottis	The space between the vocal folds, anatomically known as the rima glottidis.
Epiglottis	A cartilage flap in front of larynx that normally stands upright but close downwards to help airway protection during swallowing.
Palate	The roof of the mouth that separates the nasal and oral cavity. In mammals, the anterior portion is bony (hard palate) and the posterior portion is muscular (soft palate).
Hox genes	An evolutionary conserved group of homeobox genes that is crucial for specifying the anterior-posterior axis of an animal.
Central rhythm generator (CRG)	A neuronal circuit that produces rhythmic signals in the absence of sensory inputs. CRGs are assumed to participate in the generation of basic oropharyngeal and locomotor behaviors.

## Feeding

**Figure 1.**
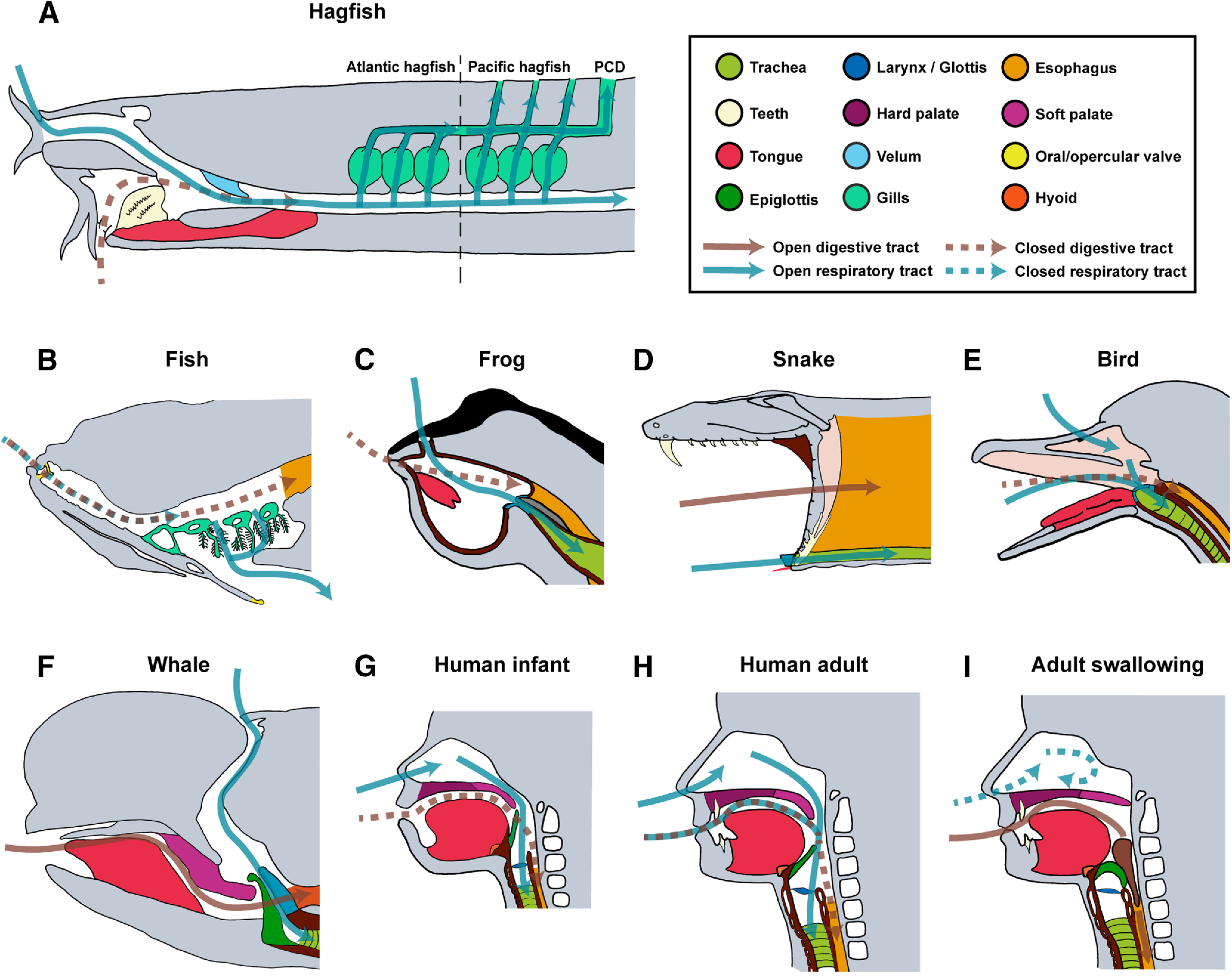
Anatomy of the oropharyngeal region involved in feeding and breathing in major vertebrate species. Schematic representation of gross oropharyngeal anatomy of different vertebrates in sagittal section. Major structures color-coded as indicated. Blue arrows indicate pathways for breathing, while brown arrows indicate pathways for feeding. ***A***, Sagittal section of a hagfish. Different gill pouches structures of Atlantic and Pacific hagfish are depicted. During breathing, the tooth plate is in its retracted position, while the velum repeatedly scrolls and unscrolls to allow water flowing in from the nostril. The water then flows through the gills and exits either at the common PCD or at individual gill slit. ***B***, Schematic sagittal view of fish oral cavity with gills depicted. ***C***, Schematic sagittal view of a frog oral cavity. ***D***, Position of a feeding snake in sagittal plane (prey is not depicted for simplicity) showing the protruded glottis. ***E***, Schematic sagittal view of a bird’s partially separated oral and nasal cavities. ***F***, Sagittal representation of an adult pilot whale (*Globicephala melaena*). The intranasal position of the larynx allows simultaneous feeding and vocalization. ***G***, Sagittal view of a human infant**. *H***, Sagittal view of a human adult. The larynx in adult is descended compared with the elevated position in infant. ***I***, Oropharyngeal structure of human adult during swallowing. The food bolus (brown) pushes the epiglottis down, allowing it to contact with the elevated larynx to assist airway protection during swallowing in adult humans. Drawings are based on or modified from Eom and Wood (2019), Ding et al. (2019), Mason et al. (2020), Cundall et al. (2014), Brown and Stallknecht (2008), Laitman and Reidenberg (1997), and Arvedson and Lefton-Greif (1998).

Since animals are unable to directly exploit solar energy, they must obtain energy from resources available in their habitat. Feeding is thus one of the primary and essential behaviors of survival. Vertebrate feeding is commonly separated into four principle stages, namely ingestion, intraoral transport, processing, and swallowing (Schwenk, 2000a). These feeding stages can be viewed as solving two major subtasks, transportation and reduction of food. The transportation process includes stages of ingestion, intraoral transport, and swallowing that move food (in original form or after reduction) from the outside environment to the esophagus, while reduction is the process of mechanically breaking down food (and this step is not needed for ingesting liquid). Vertebrates living in different environment (e.g., aquatic vs terrestrial) develop distinct oropharyngeal musculoskeletal structures and feeding mechanisms (Fig. 1; Table 2; for more details, see Bels and Whishaw, 2019).

**Table 2:** Summary of major feeding and breathing modes and their respective oropharyngeal apparatus, controller muscle groups, and putative innervating nerves

Species	Feeding/breathing modes	Oropharyngeal apparatus	Putative innervating nerves
Hagfish	Feeding	Basal plate, dentitions (palatal tooth and tooth plates), oropharyngeal and axial muscles	V (oropharyngeal muscles)
	Aquatic breathing	Velum, gill pouches	V (velum), VII, IX, X, occipitospinal nerves (gill pouches)^*^
Lamprey	Feeding, aquatic breathing	Velum (larvae), branchial basket	V (velum), VII, IX, X (branchial basket)
Fish	Feeding, forced ventilation, air-breathing	Jaw, buccal and opercular cavity (glottis, ABO for air-breathing)	V (jaw), VII (buccal and opercular cavity), IX (gill), X (gill, glottis)
	Feeding and gill ventilation	Jaw, buccal and opercular cavity	V (jaw), VII (buccal and opercular cavity), IX, X (gill)
Amphibians	Feeding	Jaw, tongue, hyoid	V (jaws), VII, IX, X (glottis)
	Air breathing	Jaw, tongue, hyoid, ABO	V (jaws), VII, IX, X (glottis), spinal nerves (abdominal muscles in expiration)
Reptiles	Feeding	Jaw, hyolingual, pharynx	V (jaws), VII, XII (hyolingual), IX, X
	Air breathing	Jaw, hyolingual, pharynx, axial muscles	V, VII, IX, X, XII, spinal nerves
Mammals	Feeding	Jaw, hyolingual, pharynx	V, VII, IX, X, XII, spinal nerves (see Table 3)
	Air breathing	Nose (hyolingual when breathing through mouth), axial muscles	VII, X, XI, XII, spinal nerves

* See Oisi et al. (2015) for more details.

### Aquatic food transportation

Food transportation in aquatic vertebrates is medium-dependent, operated mainly by producing an inward water current that pulls the food into the mouth. The most primitive form of current generation is through directional beating of cilia (no muscles involved) to produce a slow inward flow and the use of a mucus filter to capture suspended food particles (Clark and Uyeno, 2019). The evolution of branchial muscles enabled the production of larger current flows. For example, lampreys use branchial muscles to contract the branchial basket (the wall of the buccal cavity), thereby squeezing the water rapidly out of the mouth (Rovainen, 1977; Missaghi et al., 2016). The subsequent elastic (but passive) recoil of the buccal cavity brings food-bearing water into the mouth. Then water flows out through the pharyngeal slits, while food is retained and swallowed.

This primitive form of compression-followed-by-passive expansion suction evolved into a more complex sequence of active expansion-suction phases. Briefly, in most sharks and fish, feeding starts with a sudden expansion of the oropharyngeal cavity produced by rapid and nearly synchronous activation of jaw opening and branchial muscles, capturing passing preys and moving the intake caudally at high velocity (Grubich, 2001). After ingestion, through rounds of sequential moderate cavity expansions and relaxations, food is slowly transported toward the esophagus in a step-like pattern (van Meer et al., 2019; Weller et al., 2020). In addition to fish, aquatic mammals (e.g., seals and beaked whales) also secondarily evolved suction using tongue retraction and depression (Marshall et al., 2014; Kienle and Berta, 2016; Kienle et al., 2017), pointing to suction as an optimal solution for transporting prey in the aquatic environment.

Besides suction, other solutions of food transportation in the aquatic environment emerge at various stages of vertebrate evolution. For example, hagfish, which will be discussed more thoroughly in later section, repeatedly protracts and retracts the tooth plate to rip off and move the food to the esophagus (Clark et al., 2010; Zintzen et al., 2011). This cyclic pattern of protraction and retraction is aided by the posteriorly curved palatal tooth, which acts as a rachet to prevent leakage of food (Clark and Summers, 2007). On the other hand, balaenid whales constantly keep their mouths open and “swim over” dense patches of food such as copepod. The prey-laden water then flows through the baleen system, formed of structures inside the mouth that uses close-knit hair fringes called baleen hairs to filter out water and collect preys in front of the esophageal opening (Werth and Potvin, 2016; Goldbogen et al., 2017). Moreover, rorqual whales (e.g., blue whales) lunge toward and engulf a huge volume of prey-laden water during feeding. Prey items are then being filtered and transported to the esophagus mainly through hydraulic forces (Arnold et al., 2005; Werth, 2007; Werth and Ito, 2017).

### Terrestrial food transportation

In terrestrial habitats, the air’s lower density means that food transportation is no longer achievable through suction. Instead, most tetrapods (amphibians, reptiles, birds, mammals) possess a hyolingual apparatus (hyoid and tongue) that is fundamental to food transportation for these terrestrial animals. Evolution of these complex oral structures allow the animal to push or squeeze the food to the esophagus in lieu of suction.

Intraoral transport in most lizards and mammals involve repetitive cycles of tongue protrusion and retraction. Importantly, these tongue movement cycles are also tightly integrated with the cyclic jaw movements (i.e., gape cycles). Based on the velocity and direction of jaw movements, gape cycles can be divided into slow opening of the jaw, fast opening, fast closing, and slow closing-power stroke phases (Bramble and Wake, 1985; Schwenk, 2000a). During slow opening, the tongue moves anteriorly and slides under the food. The tongue and hyoid then retract back along with the food at the end of the fast opening while the jaw rapidly closes (fast closing) to prevent escape of the prey. The gape cycle then enters the last phase (slow closing-power stroke) during which food items are crushed by the teeth, which slows the jaw closing motion. Underlying mechanism of such tongue-jaw coordination is still under extensive investigations, and current results indicate contributions from both neural mechanism via common premotor neurons (Stanek et al., 2014, 2016) and mechanical linkages (for more detail, see Matsuo and Palmer, 2010).

After the coordinated actions of tongue and jaw that move the food through the oral cavity, swallowing is the final step that pushes food into the esophagus via a posteriorly-directed muscle activation sequence. Reptiles and mammals have different swallowing procedures. In many reptile species like lizards and turtles, swallowing consists of two discrete stages. First, the jaw closes and food items are being pushed to and accumulate in the pharynx in the pharyngeal packing stage by posterior and dorsal movements of the hyolingual apparatus (Schwenk and Rubega, 2005). This is then followed by the pharyngeal emptying stage that squeezes the food bolus into the esophagus by constricting the pharynx through the constrictor colli muscle (Schwenk, 2000b). In mammals, swallowing is rapidly executed within a process called deglutition because of the evolutionarily more recent invention of internal pharyngeal muscles (Schwenk, 2000a). In deglutition, the jaw is closed when the food bolus is formed. The closing of the jaw stabilizes the mandible, and the hyoid is elevated, which is accompanied by posterior tongue retraction that thrusts the food bolus posteriorly into the pharynx (Fig. 1*I*). Hyoid elevation also leads to closure of the glottis (the opening of trachea) for airway protection, i.e., preventing food from entering into the airway. As the bolus enter the pharynx, the internal pharyngeal muscles initiate a powerful descending contraction wave of the pharyngeal wall, pushing the bolus into the esophagus in a peristaltic motion (Fig. 1*I*; Ertekin and Aydogdu, 2003; Thexton et al., 2007; Miller, 2008; Lang, 2009). After the passing of food, the hyoid resumes its normal position. Together, this highly choreographed muscle activation sequence allows mammals to integrate deglutition into food reduction (called mastication, see Food Reduction) and intraoral transport cycles rather than being a discrete and prolonged kinematic stage like that in non-mammalian species (Schwenk, 2000a). Major oropharyngeal muscles, their functions, and neuronal innervations are further explained in Table 3.

**Table 3:** Major action and cranial motor nerve supply of oropharyngeal muscles in human

Muscle group	Muscle	Nerve supply	Major action
Jaw (masticatory) muscles	Masseter	V	Elevates the mandible and closes the mouth
	Temporal	V	Elevates the mandible and closes the mouth (contraction of the entire muscle); retruding the mandible (contraction of posterior fibers only)
	Medial pterygoid	V	Elevates the mandible and closes the mouth
	Lateral pterygoid	V	Protrudes and depresses the mandible (bilateral contraction); lateral excursion of the mandible to the opposite site (unilateral contraction)
Hyoid muscles (suprahyoid group)	Digastric	Anterior belly: V; Posterior belly: VII	Depresses the mandible when hyoid is fixed or elevates the hyoid and larynx if the mandible is fixed
	Mylohyoid	V	Depresses the mandible when the hyoid attachment is fixed or elevates the hyoid bone when the mandibular attachment is fixed
	Geniohyoid	C1	Assists in depression of the mandible, elevation and protrusion of the hyoid, and widening of the pharynx
	Stylohyoid	VII	Elevates and retracts the hyolingual apparatus and keeps the pharynx open during inspiration
Hyoid muscles (infrahyoid group)	Omohyoid	C2, C3	Depresses and retracts the hyoid and larynx
	Sternohyoid	C1–C3	Depresses the hyoid
	Sternothyoid	C1–C3	Depresses the hyoid and larynx when activated along with other infrahyoid muscles and opening the laryngeal inlet when activated alone
	Thyrohyoid	C1	Depresses the hyoid when activated with other infrahyoid muscles and elevates the larynx when the hyoid is stabilized
Pharyngeal muscles (outer circular layer)	Inferior constrictor	X	Constricts the wall of pharynx during swallowing
	Middle constrictor	X	
	Superior constrictor	X	
Pharyngeal muscles (inner longitudinal layer)	Stylopharyngeus	IX	Shortens and widens the pharynx during swallowing
	Salpingopharyngeus	X	
	Palatopharyngeus	X	
Tongue muscles (extrinsic)	Genioglossus	XII	Depresses and protrudes the tongue (bilateral contraction) or contralaterally deviates the tongue (unilateral contraction)
	Hyoglossus	XII	Depresses and retracts the tongue
	Styloglossus	XII	Retracts and elevates lateral portion of the tongue (midline depression known as cupping)
	Palatoglossus	X	Elevates the lingual root during swallowing and depresses the soft palate
Tongue muscles (intrinsic)	Superior longitudinal	XII	Retracts and broadens the tongue, elevates tongue apex
	Inferior longitudinal	XII	Retracts and broadens the tongue, lowers tongue apex
	Transverse	XII	Narrows and elongates the tongue
	Vertical	XII	Broadens and elongates the tongue

In addition to this hyolingual apparatus-powered mode of intraoral food transport, other land-dwelling species also evolved a diversity of methods for moving food from mouth to esophagus. For example, most frogs, crocodiles, and birds do not develop tongue and hyoid structures complicated enough to be fully responsible for hyolingual transportation. In crocodiles, the tongue lacks intrinsic musculature and is entirely connected to the mandible (Iwasaki et al., 2019). Therefore, crocodiles elevate and protrude their heads while their jaws release the food, allowing gravity along with the retracting hyoid to drop the food toward the esophagus (Cleuren and de Vree, 1992). In frogs, the root of the tongue is usually attached at the anterior portion of the mouth’s floor (Fig. 1*C*). When feeding, frogs flip out their tongues, stick to the prey, and flip back along with the prey into the mouth (Nishikawa, 2000; Herrel et al., 2019; Iwasaki et al., 2019). The subsequent swallowing is believed to be primarily driven by tongue retraction, but also substantially aided by head elevations and even retraction of eyeballs, which help push the food into the esophagus (Regal and Gans, 1976; Ritter and Nishikawa, 1995; Tso et al., 1995; Levine et al., 2004). In snakes, the jaw muscles and body both contribute to eating and swallowing preys, without assistance from the tongue, which is completely reserved for chemosensory functions (Fig. 1*D*). Using highly kinetic joints, snakes can achieve extremely large gap and cranial kinesis, providing substantial degree of freedom to the upper jaw to move relatively independently of the head. Therefore, jaws of the snake can be opened and advanced unilaterally in an alternating pattern of protraction of the upper and lower jaw on either side, functioning as the primary vector of transportation/swallowing (Moon et al., 2019). This motion is aptly termed the “pterygoid walk,” since the snake basically “walks over” the prey through a combination of ratchet-like jaw movements and concertina-like body undulation (Boltt and Ewer, 1964; Kley and Brainerd, 2002).

### Food reduction

Evolution of feeding motor programs is also dependent on food size. While animals like lampreys and balaenid whales are microphagous that transport food items in bulk, wide variety of vertebrates are macrophagous, posing the need of reduce the food into manipulatable sizes. Food reduction strategies of vertebrates, from agnathans (e.g., hagfish) to mammals, all predominantly rely on the use of teeth in both aquatic and terrestrial environment.

Macrophagy perhaps first evolved in hagfish, one of the most primitive members of existing vertebrates (Miyashita et al., 2019). The feeding apparatus of a hagfish consists of feeding musculature, a basal plate, and dentitions (tooth plates; Clark and Uyeno, 2019). The feeding musculature of hagfish are all innervated by the trigeminal nerve (Lindström, 1949) and can be divided into anterior hard component for protraction and a posterior soft component for retraction (Clark et al., 2010; Clark and Uyeno, 2019). During feeding, a hagfish repeatedly protracts tooth plates to press into the food and retracts them into the mouth along with the torn food (Clark et al., 2010). After retraction, the oral mucosa and palatal tooth dislodge the food from the teeth and transport it to the esophagus via additional protraction-retraction cycles (Clark and Summers, 2007). Because hagfish lacks the opposing jaw, to generate enough force to tear off pieces of food, the soft retractor musculature is arranged as a muscular hydrostat, which stiffens to provide a compression-resistant support (Uyeno and Clark, 2015; Clubb et al., 2019). Additionally, hagfish can twist its boneless body into a knot (a movement called body knotting) to provide further leverage (for details, see Haney et al., 2020). Thus, although hagfish is jawless, the turgid muscular hydrostat together with the body knot functions like a jaw that generates forceful bites similar to other jawed vertebrates (Clark and Summers, 2007).

In contrast (to jawless fish), mammals possess well-developed jaw apparatus. The upper and lower teeth in most mammals occlude such that they fit nicely together (Schwenk, 2000a). Occlusion within an advanced jaw enables a highly efficient form of food chewing called mastication, which allows food items to be fully comminuted because of the precise fit of the teeth. Chewing occurs mostly during the ingestion and intraoral transport stage before swallowing. The masticatory muscles for rhythmic jaw opening and closing are described in Table 3. In mammalian (e.g., sea otters) and non-mammalian gnathostomes whose teeth do not occlude, macrophagic methods like crushing and tearing become the common solution for reduction (Kienle et al., 2017). In this case, food items, usually hard-shell invertebrates, are punctured and crushed in the oral cavity so that salivary enzymes are able to further soften the food. In addition to crushing, tearing is used by carnivorous species like crocodiles, orcas, or sharks. Similar to hagfish feeding, during tearing the jaw together with the teeth grasp and rip off small pieces of food suitable for swallowing (for more details, see Bels and Whishaw, 2019).

## Breathing

Respiration/breathing is also indispensable for vertebrate survival as cells within the body need O_2_ replenishment and CO_2_ excretion to produce energy and properly metabolize. Although breathing behaviors in vertebrates all includes active muscle contractions to move O_2_ and CO_2_ in or out of the body, respiration per se is based on passive gas diffusion (Kardong, 2018). O_2_ is normally subject to a higher partial pressure in the external environment, allowing it to naturally diffuse into the blood while CO_2_ tends to diffuse out from the blood. The passive nature of gas exchanges means that all movements associated with breathing have for purpose to maintain the partial pressure gradients across the exchange surfaces, by moving gas-containing medium (water or air). Throughout evolution, many solutions for this task have emerged across different families of vertebrates, depending on the specific properties of the respiratory organ as well as the metabolic demand of the animal.

### Aquatic breathing

The emergence of active aquatic breathing involves parallel evolution of water-transporting motor programs and specialized respiration structures. Active breathing in early vertebrates is believed to be originated from feeding, partially driven by the continued evolution of pharyngeal slits (Alheid et al., 2004; Graham and Richardson, 2012; Gillis and Tidswell, 2017). In primitive chordates like amphioxus, breathing occurs mostly through the skin (cutaneous breathing; Schmitz et al., 2000; Milsom et al., 2004), and their pharyngeal slits, alongside the body, function to assist filter feeding (Graham and Richardson, 2012). As the efficiency of feeding increased because of the innovation of the muscular suction pump, the pharyngeal slits that were previously dedicated to feeding became free to take on respiratory functions. In some agnathans such as Pacific hagfish, each pharyngeal slit, also referred to as gill pouch, is separated and has its own opening to the outside, whereas the gill pouches in Atlantic hagfish are connected to a common opening called pharyngo-cutaneous duct (PCD; Fig. 1*A*). During hagfish aquatic breathing, rhythmic contraction of velum brings water into mouth through the nostril (inhalation), followed by active contractions of gill pouches to expel water out either through individual gill slits or via the common PCD (Fig. 1*A*; Malte and Lomholt, 1998; Eom and Wood, 2019). Similar motor program is also observed in premetamorphic larval lampreys (ammocoetes), in which breathing and feeding are accomplished within the same action: water enters from the mouth and exits through the pharyngeal slits by the scrolling and unscrolling action of a velum (a muscular structure that is located at the midline of pharynx), plus the compression and recoil of the branchial basket (Rovainen, 1996).

Eventually, gills are evolved to replace the pharyngeal slits in cartilaginous and teleost fish. In these species, breathing happens through gill ventilation, which uses a dual pump mechanism, namely the successive expansion and compression of the buccal and opercular cavity controlled by branchial muscles (Fig. 1*B*; Kardong, 2018; Milsom, 2018). This dual pump mechanism, generated by rostral-caudally propagating wave of brainstem motor activity (Taylor, 1992; Taylor et al., 2009b), sequentially drives the water first into the buccal cavity (buccal expansion), then across the gill curtain (buccal compression along with opercular expansion) where O_2_ diffuses passively into the gill vasculature. Finally, water containing CO_2_ to be excreted exits from the opercular valve (through opercular compression). In principle, the hydraulic nature of both fish breathing and feeding means that the same subset of cranial muscles can be used for both processes, although muscular activity during gill ventilation alone (no feeding) is slower and less powerful (Taylor et al., 2010; Milsom, 2018). Under hypoxic conditions, fish recruit additional feeding muscles to generate higher suction power to increase water flow (Taylor et al., 2006).

An important exception to the common dual pump ventilation is ram ventilation. Here, the swimming motion itself generates respiration-needed water flow across the gills, thereby saving energy costs associated to activating cranial muscles during normal gill ventilation (Roberts and Rowell, 1988). Many elasmobranchs, such as great white sharks or whale sharks, do not possess an operculum as an expiratory flap and are obligate ram ventilators that need to swim continuously for breathing (Mallatt, 1997). Other fish (e.g., nurse shark, trout) transition between gill and ram ventilation depending on swimming speed, water velocity, and/or water O_2_ tension, displaying what might be the earliest form of breathing-locomotion coordination (Randall, 1982; Steffensen, 1985).

### Air breathing

Air breathing possibly first evolved in certain bony fish species that dwell in O_2_-depleted aquatic habitats, by modifying the existing suction-based feeding-breathing mechanism. Two of the most common air-breathing organs (ABOs) are the lung and the gas bladder. The opening of ABOs to the oral cavity is gated by the glottis near the floor of digestive tract. Air-breathing fish only come out of water to breathe intermittently when they need to. During water immersion, the glottis is closed, and the cycles of buccal expansion and compression are used to move water in and out of mouth for feeding as mentioned above. During air breathing, the fish swims to the surface of the water, where the same cycles of buccal expansion and compression coupled with the opening and closing of the glottis are used for drawing air into the buccal cavity and exchanging the “old” air in the ABO with fresh air (Brainerd, 1994; Brainerd and Ferry‐Graham, 2005; Milsom, 2018). This buccal pump mechanism for breathing is retained in anuran species like frogs and toads (Fig. 1*C*; Taylor et al., 2010).

Subsequently during evolution, aspiration breathing substituted this primitive form of air breathing, with the recruitment of axial muscles for higher volume and a more efficient gas exchange. A first instance of aspiration breathing is exemplified by the traverse abdominal muscle that some salamanders use during active expiration (Brainerd, 1999). Then both inspiration and expiration became powered by axial muscles, thus drastically increasing the volume of gas exchange. For example, in lepidosaurs such as lizards and snakes, intercostal muscles are used to create a rotation of the ribs that expands the thoracoabdominal cavity and the lung (Carrier, 1989). Similar aspiration pump mechanism has been documented in birds, during which air sacs are inflated by rib rotation and caudal sternum depression (Claessens, 2004). A unique aspect of avian breathing is that within the air sac system airflow is unidirectional (Farmer, 2015). Using two aerodynamic valves, inspiring air can only enter through one valve into one set of chambers, subsequently the air moves in one direction, and expiring air leaves from the other valve back into the trachea (for more details, see Cieri and Farmer, 2016). This aerodynamic valving system maximizes breathing efficiency.

However, the reliance on axial muscles has its downsides. For instance, intercostal and abdominal muscles in lizards are also needed for locomotion. While breathing requires bilateral and synchronous activation of both muscle groups, locomotion unilaterally and alternatively activates these muscles to bend the body or to stiffen the trunk (Carrier, 1990, 1991). This functional conflict is especially evident during high-speed sprinting, during which axial muscles are solely recruited for locomotion and thus reduce the lizard’s tidal volume to near zero (Carrier, 1987). To solve this problem, many lizards, such as savannah monitor lizards, use gular (throat) pumping to circumvent the breathing-locomotion conflict. In a highly similar fashion to fish and amphibian’s buccal pumping for breathing, the gular cavity expands by retracting and depressing the hyobranchial skeleton to assist in inspiration. The addition of this non-conflicting motor program allows these lizards to draw more than two times the air volume than available through costal inspiration alone (Brainerd and Owerkowicz, 2006).

Mammals solved the respiration-locomotion conflict by evolving a novel respiratory muscle, the diaphragm, a dome-shaped muscle. Contraction of the diaphragm flattens the curvature of the dome, leading to expansion of the thoracic cavity and air influx. The diaphragm allows mammals to possess much greater stamina during locomotion, such that ventilation volume can increase along with moving speed during locomotion. Testudines (turtles and tortoises) and crocodilians also adopted similar strategies of using a dome-shaped muscle for aspiration breathing. In turtles, the internal oblique muscle contracts during inspiration, flattening the dome and increasing the thoracoabdominal cavity volume to draw air into the lung (Gaunt and Gans, 1969; Landberg et al., 2003). In crocodiles, the diaphragmaticus muscle retracts the liver that divides the thoracoabdominal cavity, thereby expanding the cavity and bringing a large volume of air into the lungs (Naifeh et al., 1970; Gans and Clark, 1976; Kardong, 2018).

## Coordination between Feeding and Breathing

Both feeding and breathing are vital to survival, and both food and O_2_ enter into the body from orofacial openings. The diverse modes of feeding and breathing through evolution also involve various mechanisms for coordinating these two behaviors.

Aquatic suction feeding and aquatic ventilation (through pharyngeal slit or gill) use many of the same muscles to bring water into the oral cavity, to push water out through pharyngeal pore/gill for gas exchange while retaining food. Thus, aquatic feeding and breathing can occur simultaneously and harmoniously as part of the same motor program. For species carrying out aquatic feeding and air-breathing, feeding (in water) and breathing (on surface of water) never overlap in time, and the glottis is normally closed to guard the lung until the time of breathing. Again, the same sets of muscles that draw water into buccal cavity are used to draw air into the mouth and the lung.

In contrast, for most tetrapods, the combination of terrestrial feeding and air breathing poses two main challenges. (1) As feeding typically takes substantial time for intraoral transportation and reduction of food, for species that depend on continuous air breathing (such as most mammals), mechanisms are needed to allow concurrent respiration and food processing (before swallowing) to occur. (2) While both air and food enter via orofacial openings, air goes to the lung, whereas food needs to enter the esophagus. Thus, air-breathing tetrapod needs to prevent food from being swallowed into the lung. Different species evolved distinct musculoskeletal elements to solve these two problems.

First, to allow concurrent breathing and food processing, structural innovations were implemented during evolution to separate the transportation pathway of air and food. For example, many snakes swallow the prey whole regardless of the size, and thus require a prolonged period of intraoral transport and slow swallowing. To enable simultaneous breathing and feeding, the snake’s oropharyngeal region has undergone significant rearrangements (Cundall et al., 2014). First, the glottis of snakes is situated at the anterior region of the oral cavity and is able to protrude outside the oral cavity during feeding to maintain breathing (Fig. 1*D*). Furthermore, the opening of the esophagus in snakes is distinctively anterior and thus effectively shortens the length of the oral cavity, such that the prey is essentially transported directly into the esophagus (Fig. 1*D*).

Another seminal structural innovation in tetropods regarding the oropharyngeal region is the development of the secondary palate (the posterior portion of hard palate and all the soft palate) that enabled the formation of a separated nasal cavity dedicated to breathing, while food only enters into the oral cavity. The secondary palatal shelves in birds or lizards are not fully fused at the midline leaving a midline cleft (Fig. 1*E*). By contrast, in mammals, crocodilians, and sea turtles, the secondary palate is completely fused at the midline, thereby fully isolating the nasal cavity from the oral cavity (Fig. 1*G*; Abramyan and Richman, 2015). This allows breathing to remain uninterrupted during the majority of food processing steps before swallowing, such as chewing and suckling in mammals or ingestion in crocodilians (who immerse their whole head except the nostrils into water during lurking).

Second, to prevent the aspiration of food into the lung during swallowing, either unique structural changes or new motor coordination programs were added. In terms of structural changes, cetaceans (whales, dolphins, and porpoises) further developed a complete segregation of their airways from the feeding passage. In these species, the larynx (entry point to trachea and lung) inserts into and completely interlocks with the nasal cavity via the palatopharyngeal sphincter (Fig. 1*F*; Reidenberg and Laitman, 1987, 1994). Food enters the oral cavity and bypasses the airway to be swallowed. Thus, cetaceans are obligatory nasal breathers and can carry out breathing/vocalization completely independent of feeding. A less extreme situation is observed in most mammals (including human infants), in which the larynx is positioned above the pharyngeal floor where food will be passed into esophagus (Negus, 1949; Laitman et al., 1977; Wolfson and Laitman, 1990; Harrison, 1995; Crompton et al., 1997; Laitman and Reidenberg, 1997). In this configuration, the epiglottis that cover the entrance of the larynx is in contact with the posterior end of the soft palate, thereby preventing the food bolus from entering the glottis (Fig. 1*G*), akin to extending a snorkeling tube above the water. This elevated position of epiglottis/larynx allows human infants to breathe and suckle milk simultaneously, and also means that most mammals are essentially nasal breathers (Moss, 1965; Laitman and Reidenberg, 1993; Maynard et al., 2020).

As human infants develop, the larynx gradually descends to the adult position (below the level of pharyngeal floor) around the second or third year (Fig. 1*H*; Lieberman et al., 2001). The descend of larynx acts as a double-edged sword: it enables a much richer vocalization repertoire, removes the obligation for nasal breathing, but also greatly increases chances of food getting into the trachea, making humans the most susceptible for food aspiration among mammalian species (Laitman and Reidenberg, 1997). To solve this problem, a reflex/motor program evolved in which swallowing temporarily suppresses breathing, with concurrent activation of muscles that close the glottis, block the nasal cavity via the soft palate, and elevate the larynx to contact the epiglottis (Fig. 1*I*).

## A hypothesis: how conserved embryonic hindbrain generates circuits for diverse modes of feeding and breathing

### Ultraconserved hindbrain and PAs during development

**Figure 2.**
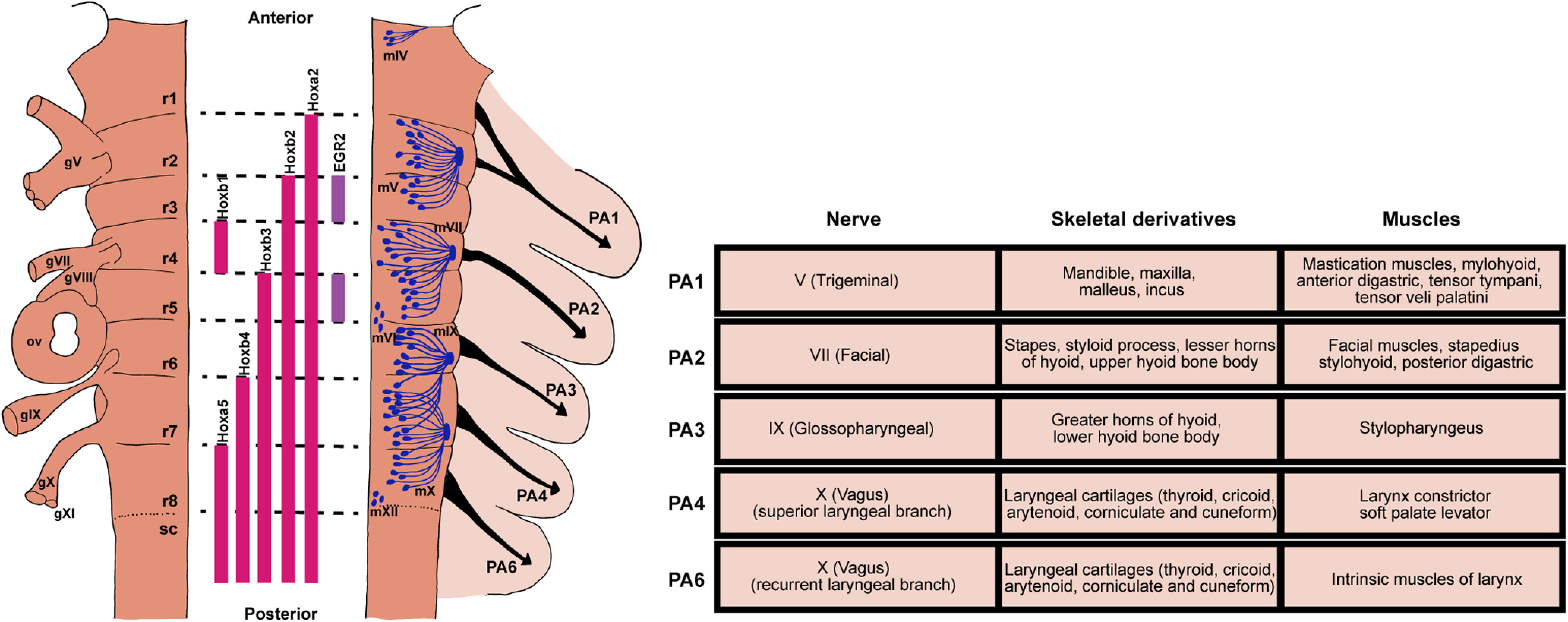
The highly conserved embryonic hindbrain and PAs and their associated circuits and peripheral structures. Left panel, Schematic representation of the vertebrate embryonic hindbrain. The developing hindbrain is segmented into rhombomeres (r1–r8), which is defined by combinatorial expression of different genes (e.g., Hox genes) and transcription factors (e.g., EGR2, also called Krox-20), depicted in the middle. Locations of cranial sensory ganglia (gV, gVII–gXI) and otic vesicles (ov) are shown on the left side of the hindbrain. The right side of the hindbrain shows motoneuron distribution of major cranial motor nuclei and their respective exit points. Neural crest cells form migratory streams (black arrows) that originate from rhombomeres to their respective PA. Right panel, Each PA is characterized by distinct nerve innervation, skeletal and muscular derivatives (table on the right). Drawings of rhombomeres and PAs and their derivatives are based on Kiecker and Lumsden (2005) and Maynard et al. (2020).

Despite the diverse modes of feeding and breathing, these two vital behaviors are controlled by neural circuits originated in the hindbrain in all vertebrates (see Fig. 2; Table 2). Importantly, all vertebrates share highly conserved embryonic developmental programs during the phylotypic period of pharyngula stage, characterized by conserved segmented structures of the embryonic hindbrain and peripheral tissues called rhombomeres (r) and PAs, respectively (Fig. 2; Irie and Kuratani, 2011; Sugahara et al., 2017; Parker et al., 2019). Each rhombomere is characterized by its unique but conserved expression of genes and transcription factors (in particular Hox and Hox-regulating genes), where specific types of motor neurons, interneurons, and neural crest cells (precursors to sensory neurons) are generated (Fig. 2; Lumsden and Keynes, 1989; Kiecker and Lumsden, 2005; Parker and Krumlauf, 2020). Each PA receives distinct yet stereotyped sensorimotor innervation and forms arch-specific musculoskeletal elements (Fig. 2). In most vertebrates, the first PA forms the jaw and is innervated by motoneurons of the Vth nerve derived from r2/r3. The second arch forms the hyoid apparatus and facial muscles and is innervated by r4/r5-derived VIIth motoneurons. The more posterior PAs either develop into gill in fish or pharyngeal and laryngeal apparatuses in tetrapods and are innervated by IXth, Xth, and XIIth motoneurons derived from r6/r7/r8 (Graham and Smith, 2001; Graham et al., 2019; Maynard et al., 2020). In other words, there is a roughly ordered anterior-to-posterior relationship between the rhombomere origins of the motor nerves and the PAs that the nerves innervate in all vertebrates. To generate diverse feeding/breathing modes based on highly conserved embryonic hindbrain and PAs, we hypothesize that initially during development, the hindbrain produces a basic rhythmic motor program common to all vertebrates, and that this early conserved circuit can be repurposed, reconfigured, or replaced during later developmental stages to generate distinct patterns of feeding and breathing. Below we consider some evidence for this hypothesis.

### A common vertebrate embryonic hindbrain circuit for rhythmic ingestion of water/amniotic fluid

**Figure 3.**
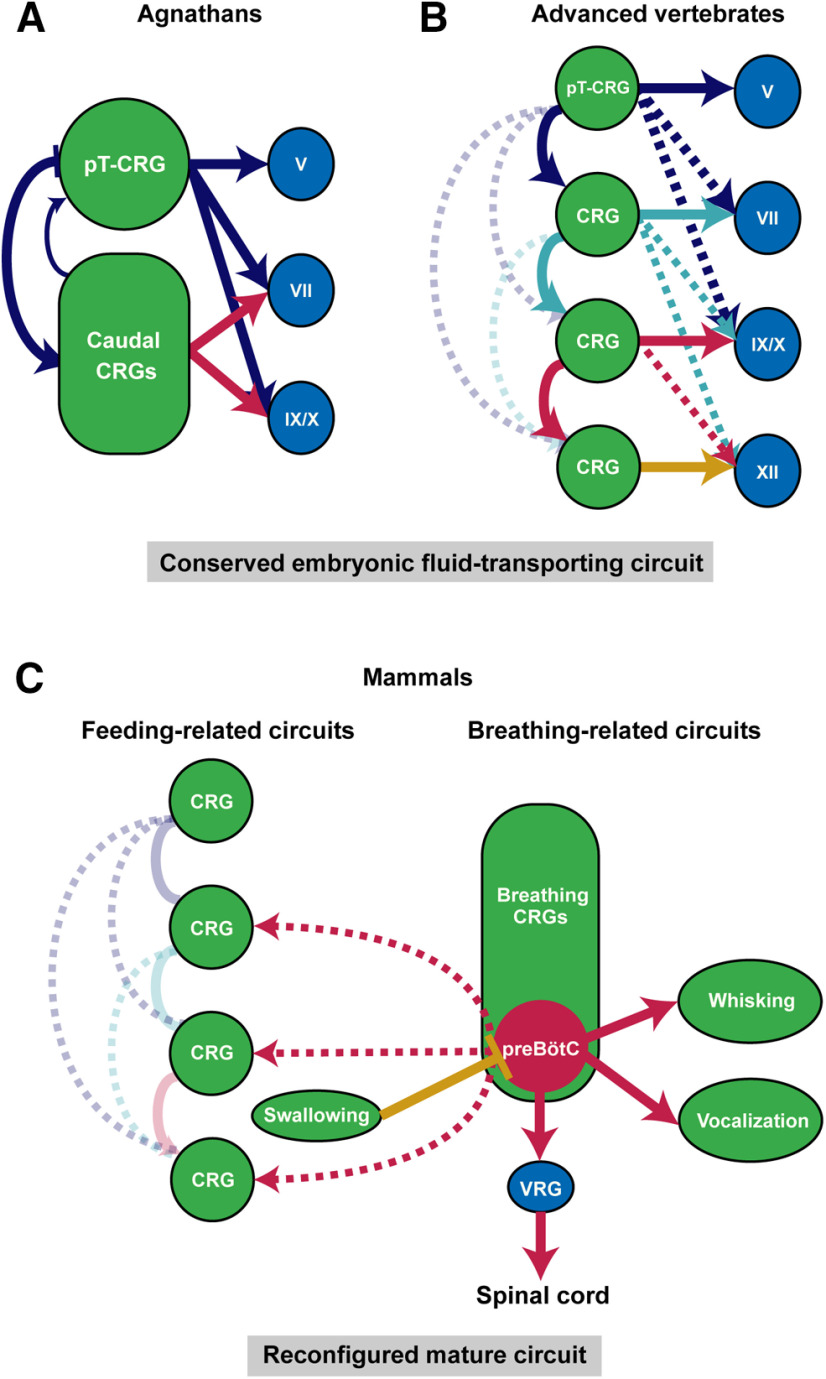
Hypothesized conserved vertebrate embryonic hindbrain circuit for intraoral fluid transportation and its later stage reconfigurations in mammals. The fluid-surrounding environment and fluid ingestion behavior in aquatic and in embryonic terrestrial vertebrates suggest the existence of a conserved embryonic circuit that generates the fluid-transporting behavior in all vertebrates (***A***, ***B***). ***A***, In primitive vertebrates like lampreys, this fluid-transporting circuit consists of two CRGs, with pT-CRG as the dominant CRG, that drive rhythmic and sequential activations of downstream motor nuclei. ***B***, In advanced vertebrates, a series of CRGs for each pair of rhombomeres work together to support the directional fluid-transporting behavior. Hypothesized serial CRGs are sequentially coupled such that an anterior CRG preferentially drives the activity of posterior CRGs, thereby produces sequential activation of oropharyngeal muscles and directional movement of fluid. ***C***, Splitting, reconfiguration and repurposing of the embryonic fluid-transporting CRGs result in separated feeding-related and breathing-related CRGs in adult mammalian hindbrain. Only preBötC is highlighted among the breathing CRGs for simplicity. The need of continuous breathing makes preBötC the dominant CRG, which broadcasts its rhythm to other CRGs or motoneurons, ventral respiratory group (VRG). Feeding-related CRGs control chewing and licking largely function independent of breathing CRGs because of structural segregations of food and air intake pathways. Swallowing inhibits breathing to prevent aspiration.

As described above, aquatic breathing and aquatic feeding share the same sets of cranial muscles and motor programs. Notably, while fish and amphibians are born directly into water, amniotes like reptiles, birds, and mammals go through embryonic development encircled by amniotic fluid. Therefore, bringing fluid into the mouth to be swallowed is likely the fundamental process common to all developing vertebrates. For example, it is known that fetal swallowing of amniotic fluid emerges right after major organogenesis around 10–14 weeks of gestation in human and is observed in early development of chicks, sheep, rodents, and monkeys (Ross and Nijland, 1998; Delaney and Arvedson, 2008; Mashayekhi et al., 2011; Gross and Trapani-Hanasewych, 2017; Maynard et al., 2020). This process of transporting fluid into the body is repeatedly executed and requires coordinated activation of oropharyngeal muscles (Sherman et al., 1990). Therefore, the developing hindbrain in all vertebrates must contains a basic circuit that produces rhythmic expansion and compression (or opening and closing) of the oral cavity for moving fluid into the mouth. We propose that this basic circuit contains either one dominant central rhythm generator (CRG) in primitive vertebrates, or a series of sequentially coupled CRGs that each has its own intrinsic rhythm in more evolved vertebrates, such that fluid is periodically transported into the mouth to be swallowed in a unidirectional manner (Fig. 3*A*,*B*).

### Existence and locations of putative fluid-transporting CRGs in developing hindbrain

Where in embryonic hindbrain might the fluid-transporting CRGs reside? Let us first consider the larval lampreys, which use rhythmic movement of the velum to pump water into the mouth for feeding and for breathing. The velum located at the midline of pharynx is innervated by the Vth nerve. During larval lamprey aquatic breathing, muscles of velum and branchial basket (innervated by VIIth, IXth, Xth motoneurons) contract repeatedly in a fixed sequence (Russell, 1986; Rovainen, 1996). This then led to the discovery of a CRG adjacent to the Vth motor nucleus termed paratrigeminal respiratory group and a distributed CRG network around VIIth, IXth, Xth motor nuclei (Fig. 3*A*; Martel et al., 2007; Missaghi et al., 2016). It is postulated that these two CRG networks are coupled such that their sequential outputs drive the repeated velar and basket pumping (and relaxation) actions to bring in water (Fig. 3*A*). Since all later jawed vertebrates (Gnathostomes) use trigeminal (V)-innervated jaw opening and closing to bring water/amniotic fluid into the oral cavity, it is likely that the CRG located initially adjacent to the Vth motor nucleus [peritrigeminal CRG (pT-CRG)] is conserved in all vertebrates (at least in developmental stages; Fig. 3*A*,*B*).

As vertebrates evolved more muscles, the lamprey-type CRG configuration may be insufficient to drive coordinated movements of many muscles used for the repeated and directional fluid transportation and swallowing processes. Unlike agnathans such as lampreys and hagfish that predominantly relies on trigeminal-innervated velum for breathing, feeding/gill ventilation in fish starts from the opening of the jaw (V), followed by the activation of branchial muscles (VII) that results in sequential expansion and compression of first the buccal and then the opercular cavity. To swallow the food into the mouth, the sequence starts after jaw closure, followed by the activation of branchial basket muscles (IX, X) that transports the food to the esophagus through rounds of oropharyngeal expansions and compression. This sequential motor pattern likely requires serially coupled-CRGs in fish (Hyde, 1904; Sundin et al., 2000; Taylor et al., 2009a). This hypothesis is further supported by “sheep-dip” experiments, in which isolated fish brainstems are gradually submerged into high magnesium solution that blocks excitatory transmission necessary for rhythmogenesis (see next section). These experiments show that rhythmic outputs of VII nerve degrade gradually rather than abruptly, indicating that the gill ventilation rhythm in fish is generated by several mutually-coupled segmental CRGs distributed throughout the hindbrain (Duchcherer et al., 2010). In the mammalian fetal swallowing process, the fluid transportation also similarly follows the sequential activation of jaw, hyolingual, and pharyngeal muscles (Sherman et al., 1990; Thexton et al., 2007).

Furthermore, evidence suggests that in higher vertebrates, each pair of rhombomeres contains its own CRG. In chick embryos, isolated hindbrain segment preparations containing either V, VII, or XII motor nuclei all exhibit spontaneous rhythmic activities that drive their respective segmental motor nerves (Fig. 3*B*; Fortin et al., 1995; Borday et al., 2003). It was also revealed that odd-even pairs of rhombomeres generate higher frequencies of rhythmic bursts (Coutinho et al., 2004). In rodent preparations, similar conclusion was drawn that rhythmic firings in V, VII, and XII motoneurons representing ingestive movements are induced by their respective segmental CRG (Nakamura et al., 1999). We further speculate that the serial CRGs in pairs of rhombomeres form directionally biased connections such that the more anteriorly located CRG preferentially drives the activity of the posteriorly located CRG, thereby facilitating the propagating wave of oropharyngeal muscle activities that moves fluid from outside of the mouth to the esophagus (Fig. 3*B*). Notably, the rhombomeres form and differentiate in a rostral-to-caudal (from r1 to r8) order (Gavalas, 2002). Thus, the serial CRGs may also follow a developmental sequential manner of maturation thereby allowing the anterior CRGs to preferentially drive the posterior CRGs (Fig. 3*B*). The sequential coupling of CRGs is also likely dependent on and facilitated by the developing sensory neurons that form feedback circuits in favor of sequential activation. In isolated whole embryonic hindbrain preparations, it was shown that all cranial motor nerves exhibit synchronized rhythmic activities (Fortin et al., 1995; Abadie et al., 2000), suggesting that in the absence of sensory feedback, a single CRG (likely the most anterior pT-CRG) dominates.

### Evolutionary conserved characteristics of rhythm-generating mechanism

Investigations of rhythm-generating mechanisms behind oropharyngeal behaviors in vertebrates have largely focused on breathing, especially in lampreys, frogs, and rodents. These studies primarily examined the roles of excitatory glutamatergic mechanisms, inhibitory GABAergic and glycinergic mechanisms, putative pacemaker properties, and neuromodulators in rhythm generations. Decades of studies suggest that some rhythmogenic mechanisms are evolutionary conserved.

First, excitatory glutamatergic system is essential for rhythm generation in nearly all vertebrate breathing CRGs. Both lamprey’s pT-CRG and mammalian’s inspiration CRG, preBötzinger complex (preBötC), contain large ensembles of glutamatergic neurons and are sensitive to modulations by opioids and substance P (Mutolo et al., 2010; Del Negro et al., 2018). In lampreys, microinjections of AMPA and NMDA blockers in pT-CRG temporary abolish its rhythm (Martel et al., 2007; Cinelli et al., 2013). On the other hand, manipulations that facilitate glutamatergic transmission (e.g., application of AMPA or perfusion of mGluRs I and II agonists) accelerate pT-CRG rhythm (Bongianni et al., 2002; Martel et al., 2007; Cinelli et al., 2013). In mammals, many neurons that express transcription factor developing brain homeobox protein 1 (DBX1) during the pharyngula stage later become glutamatergic neurons, including neurons in preBötC. Dbx1-knock-out mice do not breathe and die at birth and optogenetic inhibition of DBX1 lineage cells slows and stops breathing rhythm (for more detail, see Del Negro et al., 2018). Glutamatergic transmission is also found to be essential in CRGs responsible for lung ventilation in turtles (Johnson and Mitchell, 1998) and frogs (Chen and Hedrick, 2008; Baghdadwala et al., 2016). Interestingly, recent results from Ashhad and Feldman (2020) suggest that inspiratory rhythmogenesis in neonatal mammalian preBötC is critically driven by emergent network properties, where strong synchronization among excitatory rhythmogenic neurons within preBötC produces inspiratory bursts.

Second, inhibitory systems heavily modulate the frequency and amplitude of rhythm but may not be essential for rhythm generation per se. For example, respiratory rhythm still persists after blockade of GABAergic and glycinergic transmission in lampreys (Bongianni et al., 2006; Cinelli et al., 2014, 2016), in frogs (lung ventilation; Leclère et al., 2012; Baghdadwala et al., 2016), turtles (Johnson et al., 2002, 2007), and in mammals (Janczewski et al., 2013; Sherman et al., 2015; Baertsch et al., 2018). However, in different species or different brainstem nuclei, inhibition has different effects. Blocking GABA_A_ in lamprey produce significant increases in frequency and amplitude of breathing rhythm, while injection of GABA_A_ agonist suppresses pT-CRG rhythm (Bongianni et al., 2006; Cinelli et al., 2014). In rodents, it is proposed that GABAergic and glycinergic transmissions, respectively, regulate different aspects of rhythm and pattern generated by preBötC (Ashhad and Feldman, 2020). Photostimulation of glycinergic preBötC neurons depresses breathing while inhibition of these neurons augments breathing amplitude and stops ongoing apnea (Sherman et al., 2015). Additionally, complex reciprocal GABAergic transmissions are important for the coupling of inspiratory preBötC and expiratory parafacial oscillator (Mellen and Thoby-Brisson, 2012). By contrast, in chick embryos, blocking GABA-mediated inhibition in segmental CRGs abolishes the high frequency rhythmic bursts. Here, inhibition is required for generating high (but not low) frequency rhythms (Fortin et al., 1999).

Many studies also examined whether pacemaker-like properties are involved in hindbrain rhythmogenesis across species. Specifically, these studies tested the roles of persistent sodium current (*I*_NaP_) or calcium-activated non-specific cation (*I*_CAN_) currents. In lampreys, turtles, and mammals, while breathing rhythm is abolished by *I*_NaP_ and *I*_CAN_ current blockers, application of an exogenous excitatory agent (e.g., substance P) restores the rhythm (Del Negro et al., 2005; Mutolo et al., 2010; Lin and Onimaru, 2015; Johnson et al., 2016). These results suggest that pacemaker properties are not essential for respiratory rhythm generation (Mellen and Thoby-Brisson, 2012; Bongianni et al., 2016; Missaghi et al., 2016). However, under hypoxia condition, breathing-related activity are dependent on *I*_NaP_ (Peña et al., 2004; Paton et al., 2006). Furthermore, it was shown that in mouse, unlike the primarily network-driven rhythmogenesis at and after E18.5, *I*_NaP_ and *I*_CAN_ dependent pacemaker properties are purely required for inspiration rhythm in embryos before E16.5 (Chevalier et al., 2016). Thus, the rhythmogesis mechanisms for the proposed fluid-transporting CRGs in earlier embryos could be different from mechanisms governing CRGs in neonatal and adult higher vertebrates, and future studies with cellular identities are needed to pinpoint the exact mechanisms.

### Reconfiguration and repurposing embryonic CRGs to accommodate parallel air breathing and feeding in higher vertebrates

The evolutionary progression from one oral cavity serving both feeding and breathing functions in aquatic vertebrates, to two separated food/fluid and air intake pathways, such as the separated nasal and oral cavities in mammals, allows air-breathing and feeding to be conducted, to a large extent, independently. We hypothesize that peripheral structural separations are accompanied by the split of conserved segmental fluid-transporting CRGs in the hindbrain into two separate sets: one set for breathing and the other set remains as CRGs for feeding (Fig. 3*C*).

Indeed, a series of breathing-related CRGs have been discovered in mammals. They are, from rostral to caudal, the parafacial respiratory group functioning as the expiratory CRG (pFRG), the postinspiration complex (PiCo) hypothesized to be the postinspiration CRG, and the preBötC known as the inspiratory CRG. These respiratory CRGs may have migrated during development from their original birthplace to their final ventral locations in the hindbrain. We speculate that the origin of preBötC might be the segmental CRG near the Xth nerve (in r7) that is recruited during evolution to provide rhythmic control of inspiration axial muscles (by projecting to neurons in the medullary ventral respiratory groups which in turn project to the spinal cord). Interestingly, it was shown in mouse, that the constituent neurons of the embryonic parafacial (e-pF; originated in r4) and preBötC (in r7) CRGs are born and matured in a sequential fashion [e-pF cells are born at embryonic day (E)10.5 and matured at E14.5, while preBötC neurons are born at E11.5 and matured at E15.5], allowing the anterior-located e-pF to entrain the immature preBötC activity in early embryonic stages (Thoby-Brisson et al., 2009; Champagnat et al., 2011). This time period also coincides with the period of secondary palate formation (Yu et al., 2017). After the complete fusion of the secondary palate at the midline to separate nasal and oral cavity in late embryonic stages, preBötC gradually becomes the dominant CRG in the hindbrain, entraining other respiratory CRGs (Ramirez et al., 2016; Del Negro et al., 2018), perhaps in preparation for the need of continuous air-breathing after birth. In addition, other secondary CRGs serve orofacial actions using muscles evolutionarily involved in breathing may be further separated/differentiated or repurposed from embryonic ones. For example, parts of posterior embryonic CRG neurons in r7/r8 are likely to be repurposed as CRGs for whisking in rodents and vocalizations in mammals (Fig. 3*C*).

On the other hand, some of the embryonic fluid-transporting CRG neurons retain their feeding-related functions. These cells may stay in their conserved locations near feeding-related motor neurons. For example, the ancient pT-CRG may become the mammalian CRG for suckling (neonates) and chewing (adults). Indeed, neurons located dorsal to and near Vth nucleus are implicated in controlling rhythmic jaw movements (Morquette et al., 2012). The caudal feeding-related embryonic CRGs likely further differentiated to control licking and swallowing (Fig. 3*C*). Again, because of the separation of nasal and oral cavity, chewing and licking can operate to a large extent independently of breathing until the step of swallowing in most mammals (Moore et al., 2014). On the other hand, the continuously active inspiration CRG preBötC is shown to send strong projections throughout the hindbrain, broadcasting the breathing rhythm to neurons controlling feeding (Fig. 3*C*). This is likely because breathing requires activity from certain feeding-related oropharyngeal muscles to help maintain airway patency (Moore et al., 2014; Yang and Feldman, 2018). Finally, swallowing in mammals is typically executed is one rapid motion and thus it may not require a CRG. However, new airway protection circuits must have evolved in mammals such that centrally-arisen or peripherally-arisen swallowing signals effectively inhibit inspiration to prevent aspiration of food (Fig. 3*C*).

The maturation and refinement of species-specific hindbrain orofacial circuits likely depends on feedback signaling from peripheral muscles and other tissues to motoneurons, then from motoneurons to upstream CRGs and other interneurons also through retrograde signaling, as well as depends on activity of sensory feedback. Gradually, synergistic muscles will be co-active in the same phase of CRG rhythm, whereas antagonist muscles will be co-activated in the opposite phase of CRG activity. Sequences are further consolidated and eventually diverse motor patterns of coordinated feeding and breathing behaviors in higher vertebrates emerge.

## Concluding remarks

One of the core questions related to the development and evolution of motor behaviors is how evolutionary conserved developmental principles give rise to such a diverse pool of behaviors. In this review, we discussed how vertebrates, while carrying out varied modes of feeding and breathing, all share a stage of living in a fluid and all execute a homologous action of periodically transporting fluid from the external environment inside the body. We hypothesize that a conserved hindbrain circuit generating a posteriorly propagating rhythmic activity is initially formed in all vertebrates, that drives the basic intraoral fluid transport behaviors. This circuit forms the basis for carrying out aquatic feeding/aquatic breathing, primitive air breathing, and terrestrial food transportation. Aspiration breathing and food reduction (chewing) represent the splitting, repurposing, and reconfiguration of the existing hindbrain CRGs. We note that other than preBötC/pF/PiCo, the neuronal identities and exact locations of feeding-related CRGs (chewing, suckling, licking, swallowing, etc.) in mammals remain vague. Based on our unifying hypothesis, we propose that studies of hindbrain CRGs for feeding/gill ventilation in fish may reveal the homologous neurons in mammals that drive rhythmic suckling, chewing, and licking. Alternatively, if a new method can be developed that enables lineage tracing of hindbrain CRG neurons in mammalian embryos, following these neurons into postnatal development and adult should provide insights for the locations and identities of adult CRG neurons.

## Synthesis

Reviewing Editor: Lynda Erskine, University of Aberdeen Decisions are customarily a result of the Reviewing Editor and the peer reviewers coming together and discussing their recommendations until a consensus is reached. When revisions are invited, a fact-based synthesis statement explaining their decision and outlining what is needed to prepare a revision will be listed below. The following reviewer(s) agreed to reveal their identity: Theodore Uyeno.

Thank you for submitting your article to eNeuro which has been reviewed by 2 experts in the field. We are agreed that your review article presents a really integrative conceptual analysis that attempts to bring together the mechanics of organisms breathing and feeding in various media, the control of these behaviors, and the possible evolution of their neural control. However, following consultation between the reviewers and myself, we are agreed that there are several areas where the article could be strengthened prior to publication. In particular, we are agreed that the article should address in much more detail the similarities and differences described so far between the hindbrain rhythm generating circuitries for different behaviors across different species.

Below is a synthesis of the points from both reviewers that we are all agreed should be addressed.

Major points:

1. Only about a quarter of the review is devoted to neural mechanisms underlying feeding and breathing. The rhythm generating mechanisms and circuitry are superficially addressed in comparison to the first part of the review. A more robust comparative analysis of the rhythm generating circuitries is required.

2. The first and largest part of the review is devoted to the description of feeding and breathing behaviors and the constraints imposed by the conditions in which the animals evolve. Some phases of these movements are described in detail (such as for transporting food, or the mechanisms in place to prevent food aspiration), while others, such as reducing food movements, are barely addressed.

3. Here the authors are trying to bring together understanding of neural control with both comparative anatomy and functional/mechanical processes (breathing and feeding behaviors) in order to inform on the evolutionary modes and processes of vertebrates. With the presentation of their hypothesis, they even point out future experimental possibilities. There is real value to literature reviews if they are rigorous and complete. I think this manuscript is a great start, but omits a lot of details of the agnathans, and especially the hagfish, which consensus suggests is a vertebrate (e.g. Miyashita et al, 2019 PNAS). Including more details on the early evolution of vertebrates can only help strengthen this paper (see points 4, 15, 17 and 25 below).

4. With respect to the "Aquatic food transportation" and following sections, I think you’re missing a whole section on the potentially very interesting agnathans. With the idea that hagfish are vertebrates now being pretty solid, I think addition of this material would be useful in a review. They have a very interesting food processing/transport mechanism (Clubb Clark Uyeno, 2019) that is used in conjunction with knotting to offset the lack of jaws (Haney Clark Uyeno 2019) in order to feed on carrion and live prey (Zintzen et al. 2011). There is even something known about their brains (Sugahara et al. 2017). I think this is important because it presumably informs on the initial state of "Feeding and breathing of vertebrates through evolution".

Other points:

5. The steplike pattern described in the second paragraph of the aquatic food transport section is not obvious in figure 1A.

6. The title was found to be confusing, consider "Vertebrate evolution conserves circuits despite diverse feeding and breathing modes".

7. As a review paper, adding references to the introductory paragraph would be useful to create access to the basic literature.

8. Page 3 3rd paragraph. The phrasing of the sentence (and "swim over" dense patches of prey

like copepod) is awkward. Copepods are prey like?

9. Same sentence: (through the baleen system, a structure inside). Consider (through the baleen system, formed of structures inside)

10. Page 4 2nd paragraph. food transportation is no longer achievable through suction. It would be useful to mention the terrestrial alternative to suction in this paragraph.

11. Page 4 last paragraph. (After the coordinated actions of tongue and jaw that move the food through the oral cavity,) These actions are postulated to be neural, but can you review possible hypotheses of mechanical linkages?

12. Page 5 1st paragraph. Consider: (In deglutition, the jaw is closed.. otherwise there is no link between the term and its following definition.

13. Page 6. 1st paragraph. (retracting hyoid to drag the food towards the esophagus) It would more be drop than drag wouldn’t it?

14. same paragraph. (the jaw becomes the primary vector of transportation). I don’t think conveys the rather extreme cranial kinesis that occurs in whole snake heads while ingesting large prey items.

15. Page 6 2nd paragraph. (nonmammalian species whose teeth do not occlude, crushing and tearing becomes the common solution). This is true for hagfish and lamprey as well. They represent animals that have teeth that not only don’t occlude, they don’t even have teeth that oppose!

16. Page 7 2nd paragraph. (Oxygen normally subject to a higher partial pressure). You’re missing a ver here.

17. Page 7 3rd paragraph. (For primitive chordates, breathing occurs mostly through the skin). Which primitive chordates? And again, it may be useful to summarize both hagfish and lamprey here. Hagfish have a single naris and they both have that unique velum organ. There is some wonderful diversity in hagfish with connected (myxine) and separate (eptatretine) gill pouches.

18. Page 9 2nd paragraph. (are used for drawing air into mouth and exchange the "old" air) I’m not sure of the reference, but isn’t this referred to as positive pressure ventilation?

19. Page 10 1st paragraph. (Similar aspiration pump mechanism has been documented in birds, during) It could be worth it to note that birds use that weird, but highly efficient one-way flow system based on dynamic valving.

20. Page 11 3rd paragraph. (used to draw air into the mouth and the ABO.) I don’t like acronyms in general and this one, I’m concerned causes the reader to consider the respiration-dedicated lung with the vascularized air buoyancy bladder as equal in those that have both... many considered here do not.

21. Page 15 2nd paragraph. (Where in embryonic hindbrain might the fluid-transporting CRG or serial CRGs reside?) What is the distinction between a CRG attached to a distributed network of CRGs and a series of CRGs? It wasn’t very clear in comparing this, and the next, section.

22. Page 16 2nd paragraph (the lamprey-type CRG configuration) What would the telltale evidence be to show insufficiency in the lamprey type CRG system? It could use a more direct comparison of the two systems in order to better gauge the import of the change. Can you briefly describe the increasing evidence that gill ventilation rhythm is generated by segmental CRGs?

23. Page 17 2nd paragraph. (oral cavities as described above,) In which animals?

24. Figure 1. This figure requires more complete annotation. Not all the letters are described in the text. Some food and air paths are dashed lines while others are not.

25. Table 2. Since you list the innervating nerves as putative, you can definitely add Hagfish to this table.

## Author Response

Dear Dr. Erskine,

Thank you and the reviewers for the highly constructive review of our manuscript. We have now

thoroughly addressed these comments. Please see below for point-by-point responses.

Synthesis of Reviews:

Synthesis Statement for Author (Required):

Thank you for submitting your article to eNeuro which has been reviewed by 2 experts in the

field. We are agreed that your review article presents a really integrative conceptual analysis

that attempts to bring together the mechanics of organisms breathing and feeding in various

media, the control of these behaviors, and the possible evolution of their neural control.

However, following consultation between the reviewers and myself, we are agreed that there are

several areas where the article could be strengthened prior to publication. In particular, we are

agreed that the article should address in much more detail the similarities and differences

described so far between the hindbrain rhythm generating circuitries for different behaviors

across different species.

Thank you for the positive comment that our review is an "integrative conceptual analysis".

Below is a synthesis of the points from both reviewers that we are all agreed should be

addressed.

Major points:

1. Only about a quarter of the review is devoted to neural mechanisms underlying feeding and

breathing. The rhythm generating mechanisms and circuitry are superficially addressed in

comparison to the first part of the review. A more robust comparative analysis of the rhythm

generating circuitries is required.

We thank the reviewers for pointing this out. We added a new sub-section in the manuscript

("Evolutionary conserved characteristics of rhythm-generating mechanism") that reviews

literatures on rhythm-generating mechanisms, specifically focusing on comparing and

summarizing evolutionary conserved aspects. In addition, we also elaborated possible rhythmgenerating mechanism of fluid-transporting CRGs based on the literature we reviewed.

2. The first and largest part of the review is devoted to the description of feeding and breathing

behaviors and the constraints imposed by the conditions in which the animals evolve. Some

phases of these movements are described in detail (such as for transporting food, or the

mechanisms in place to prevent food aspiration), while others, such as reducing food movements,

are barely addressed.

Agreed. We have added a full paragraph summarizing hagfish feeding in the food reduction

section, which includes an in-depth description of hagfish feeding with tooth plates and the usage

of its jawless oral apparatus and body knotting to facilitate tearing of food.

3. Here the authors are trying to bring together understanding of neural control with both

2

comparative anatomy and functional/mechanical processes (breathing and feeding behaviors) in

order to inform on the evolutionary modes and processes of vertebrates. With the presentation of

their hypothesis, they even point out future experimental possibilities. There is real value to

literature reviews if they are rigorous and complete. I think this manuscript is a great start, but

omits a lot of details of the agnathans, and especially the hagfish, which consensus suggests is a

vertebrate (e.g. Miyashita et al, 2019 PNAS). Including more details on the early evolution of

vertebrates can only help strengthen this paper (see points 4, 15, 17 and 25 below).

We thank the reviewers pointing us to the consensus now that hagfish is a vertebrate. As

mentioned above, we have added an in-depth summary on hagfish feeding in aquatic food

transportation (several sentences in page 3-4) and the food reduction section (one paragraph on

page 7), as hagfish feeding might represent the primitive vertebrate macrophagy mechanism. We

also added a hagfish diagram in Figure 1 to give a clear depiction of hagfish oropharyngeal

structures. In addition, we also mentioned hagfish in other appropriate sentences, especially

sections discussing aquatic modes of breathing of vertebrates (several sentences on page 9).

4. With respect to the "Aquatic food transportation" and following sections, I think you’re

missing a whole section on the potentially very interesting agnathans. With the idea that hagfish

are vertebrates now being pretty solid, I think addition of this material would be useful in a

review. They have a very interesting food processing/transport mechanism (Clubb Clark Uyeno,

2019) that is used in conjunction with knotting to offset the lack of jaws (Haney Clark Uyeno

2019) in order to feed on carrion and live prey (Zintzen et al. 2011). There is even something

known about their brains (Sugahara et al. 2017). I think this is important because it presumably

informs on the initial state of "Feeding and breathing of vertebrates through evolution".

Yes, we totally agree with this comment. In addition to adding descriptions of hagfish feeding

processes, we also added detailed description of breathing modes of different hagfish species

(Atlantic versus Pacific), and compare them with other vertebrates from an evolutionary

perspective. We also expanded Figure 1 to include two different kinds of hagfish species.

Other points:

5. The steplike pattern described in the second paragraph of the aquatic food transport section is

not obvious in figure 1A.

We thank the reviewers for the suggestion. While it is difficult to depict the sequential breathing

steps in one figure, we opt to show the step where water flows out from the gill. Briefly, we

showed the oral valve closed (after water intake) while we showed the expanded operculum and

opening the opercular valve. Therefore, Figure 1B (previously Figure 1A) now depicts the stage

during which the fish expands its operculum and closes its oral valves to push the water through

the gills. We also deleted the sentence stating Figure 1B shows the breathing steps to avoid

confusion.

6. The title was found to be confusing, consider "Vertebrate evolution conserves circuits despite

diverse feeding and breathing modes".

3

We thank the reviewers’ suggestion on the title. We have changed the title of the review to

"Vertebrate evolution conserves hindbrain circuits despite diverse feeding and breathing modes".

7. As a review paper, adding references to the introductory paragraph would be useful to create

access to the basic literature.

Agreed. We have now cited relevant literatures in the Introduction, as well as in introductory

paragraphs for feeding and breathing.

8. Page 3 3rd paragraph. The phrasing of the sentence (and "swim over" dense patches of prey

like copepod) is awkward. Copepods are prey like?

We apologize for the confusion. We have changed "prey like" to "food such as" to avoid

potential confusion to the readers.

9. Same sentence: (through the baleen system, a structure inside). Consider (through the baleen

system, formed of structures inside)

We have changed the phrase according to the suggestion.

10. Page 4 2nd paragraph. food transportation is no longer achievable through suction. It would

be useful to mention the terrestrial alternative to suction in this paragraph.

Based on the reviewers’ suggestion, we added a sentence at the end of paragraph that mentioned

major terrestrial alternatives to suction.

11. Page 4 last paragraph. (After the coordinated actions of tongue and jaw that move the food

through the oral cavity,) These actions are postulated to be neural, but can you review possible

hypotheses of mechanical linkages?

Based on the reviewers’ suggestion, we cited references that examines the potential neural

mechanisms and mechanical linkages (Matsuo & Palmer, 2010) to tongue-jaw coordination at

the end of the previous paragraph.

12. Page 5 1st paragraph. Consider: (In deglutition, the jaw is closed.. otherwise there is no link

between the term and its following definition.

We have fixed the sentence according to the suggestion.

13. Page 6. 1st paragraph. (retracting hyoid to drag the food towards the esophagus) It would

more be drop than drag wouldn’t it?

We have changed the wording from "drag" to "drop".

14. same paragraph. (the jaw becomes the primary vector of transportation). I don’t think

4

conveys the rather extreme cranial kinesis that occurs in whole snake heads while ingesting

large prey items.

The reviewers are absolutely correct regarding the importance of cranial kinesis on snake feeding.

We added a sentence "Utilizing highly kinetic joints, snakes can achieve extremely large gap and

cranial kinesis, providing substantial degree of freedom to the upper jaw to move relatively

independently of the head".

15. Page 6 2nd paragraph. (nonmammalian species whose teeth do not occlude, crushing and

tearing becomes the common solution). This is true for hagfish and lamprey as well. They

represent animals that have teeth that not only don’t occlude, they don’t even have teeth that

oppose!

The reviewers are absolutely correct regarding this issue. Together with suggestions from the

major points, we’ve added a detailed description on how food reduction is executed in agnathans

using hagfish as an example. We also changed "species" to "gnathostomes" to avoid confusion

and repetition.

16. Page 7 2nd paragraph. (Oxygen normally subject to a higher partial pressure). You’re

missing a verb here.

We added a verb to this sentence: "Oxygen is normally subject to a higher partial pressure in the

external environment..".

17. Page 7 3rd paragraph. (For primitive chordates, breathing occurs mostly through the skin).

Which primitive chordates? And again, it may be useful to summarize both hagfish and lamprey

here. Hagfish have a single naris and they both have that unique velum organ. There is some

wonderful diversity in hagfish with connected (myxine) and separate (eptatretine) gill pouches.

We thank the reviewers for pointing these out. We added "amphioxus" in the sentence to avoid

confusion. We also added several paragraphs that summarized how aquatic breathing was

initially evolved, as well as why studying major breathing strategies and structures in both

hagfishes and lampreys can provide valuable insights on breathing evolution. In addition, we

also updated our Figure 1 to include a diagram of both Atlantic and Pacific hagfish, showing the

structure of connected and separate gill pouches (Figure 1A).

18. Page 9 2nd paragraph. (are used for drawing air into mouth and exchange the "old" air) I’m

not sure of the reference, but isn’t this referred to as positive pressure ventilation?

We think "positive pressure ventilation" is used to refer to a respiratory therapy that delivers air

into the lung alveoli via positive pressure and passively expires the air out from pressurized

alveoli to less pressurized conductive airways (Potchileev et al., 2020). However, to avoid

confusion, we modified the sentence to explicitly state that air is drawn in due to the negative

pressure created by expansion of buccal cavity and opening of the glottis.

19. Page 10 1st paragraph. (Similar aspiration pump mechanism has been documented in birds,

5

during) It could be worth it to note that birds use that weird, but highly efficient one-way flow

system based on dynamic valving.

We thank the reviewers for pointing out this interesting body of literatures. We have added

several sentences following the bird breathing to summarize how bird utilized aerodynamic

valving and unidirectional breathing to achieve efficient gas exchange.

20. Page 11 3rd paragraph. (used to draw air into the mouth and the ABO.) I don’t like

acronyms in general and this one, I’m concerned causes the reader to consider the respirationdedicated lung with the vascularized air buoyancy bladder as equal in those that have both...

many considered here do not.

We have replaced "ABO" with "lung".

21. Page 15 2nd paragraph. (Where in embryonic hindbrain might the fluid-transporting CRG

or serial CRGs reside?) What is the distinction between a CRG attached to a distributed network

of CRGs and a series of CRGs? It wasn’t very clear in comparing this, and the next, section.

The reviewers are correct that this is somewhat vague distinction between CRG attached to a

distributed network versus a series of CRGs. Basically, CRG of a distributed network shares one

rhythm and neurons fire in synchrony, whereas a series of CRGs that are coupled could have

different rhythms when they are in isolation. In the manuscript, we added citations for serial

CRGs: "This sequential motor pattern likely requires serially coupled-CRGs in fish (Hyde, 1904;

Sundin et al., 2000; Taylor et al., 2009a)".

22. Page 16 2nd paragraph (the lamprey-type CRG configuration) What would the telltale

evidence be to show insufficiency in the lamprey type CRG system? It could use a more direct

comparison of the two systems in order to better gauge the import of the change. Can you briefly

describe the increasing evidence that gill ventilation rhythm is generated by segmental CRGs?

Yes, we have added direct comparison between the motor program complexity between

agnathans and fish. In addition, we also described the major experiment and literatures that leads

to the conclusion of segmental CRGs behind gill ventilation: "This hypothesis is further

supported by "sheep-dip" experiments, in which isolated fish brainstems are gradually

submerged into high magnesium solution that blocks excitatory transmission necessary for

rhythmogenesis (see below). These experiments show that rhythmic outputs of VII nerve

degrade gradually rather than abruptly, indicating that the gill ventilation rhythm in fish is

generated by several mutually-coupled segmental CRGs distributed throughout the hindbrain

(Duchcherer et al., 2010)."

23. Page 17 2nd paragraph. (oral cavities as described above,) In which animals?

We added "in mammals" to clarify the species.

24. Figure 1. This figure requires more complete annotation. Not all the letters are described in

the text. Some food and air paths are dashed lines while others are not.

6

We expanded the color-coding references and described all of the relevant structures and dashed

versus solid lines in the figure.

25. Table 2. Since you list the innervating nerves as putative, you can definitely add Hagfish to

this table.

Yes. We have added hagfish to the Table. Thank you!

We are really grateful for all the detailed and constructive comments. We hope this revised

manuscript now is suitable for publication in eNeuro.
